# Protective Effects of Acyl-coA Thioesterase 1 on Diabetic Heart via PPARα/PGC1α Signaling

**DOI:** 10.1371/journal.pone.0050376

**Published:** 2012-11-30

**Authors:** Shenglan Yang, Chen Chen, Hong Wang, Xiaoquan Rao, Feng Wang, Quanlu Duan, Fuqiong Chen, Guangwen Long, Wei Gong, Ming-Hui Zou, Dao Wen Wang

**Affiliations:** 1 Department of Internal Medicine and Gene Therapy Center, Tongji Hospital, Tongji Medical College of Huazhong University of Science and Technology, Wuhan, P. R. China; 2 Department of Medicine and Endocrinology, University of Oklahoma Health Science Center, Oklahoma City, Oklahoma, United States of America; University of Western Ontario, Canada

## Abstract

**Background:**

Using fatty acids (FAs) exclusively for ATP generation was reported to contribute to the development of diabetic cardiomyopathy. We studied the role of substrate metabolism related genes in the heart of the diabetes to find out a novel therapeutic target for diabetic cardiomyopathy.

**Methods and Results:**

By microarray analysis of metabolic gene expression, acyl-CoA thioesterase 1 (acot1) was clearly upregulated in the myocardia of db/db mice, compared with normal control C57BL/Ks. Therefore, gain-of-function and loss-of-function approaches were employed in db/db mice to investigate the functions of ACOT1 in oxidative stress, mitochondrial dysfunction and heart function. We found that in the hearts of db/db mice which overexpressed ACOT1, H_2_O_2_ and malondialdehyde (MDA) were reduced, the activities of ATPases in mitochondria associated with mitochondrial function were promoted, the expression of uncoupling protein 3 (UCP3) contributing to oxygen wastage for noncontractile purposes was decreased, and cardiac dysfunction was attenuated, as determined by both hemodynamic and echocardiographic detections. Consistently, ACOT1 deficiency had opposite effects, which accelerated the cardiac damage induced by diabetes. Notably, by real-time PCR, we found that overexpression of ACOT1 in diabetic heart repressed the peroxisome proliferator-activated receptor alpha/PPARγ coactivator 1α (PPARα/PGC1α) signaling, as shown by decreased expression of PGC1α and the downstream genes involved in FAs use.

**Conclusion:**

Our results demonstrated that ACOT1 played a crucial protective role in diabetic heart via PPARα/PGC1α signaling.

## Introduction

Cardiovascular complications associated with diabetes are leading causes of morbidity and mortality of diabetic patients [1].

Diabetic cardiomyopathy is defined as reduced or low-normal left ventricular function that occurs independent of coronary artery disease and hypertension in type 1 or type 2 diabetic patients [2], [3], [4]. Under normal conditions, fatty acids (FAs) and glucose are used as the main energy substrates to generate ATP for cardiac work [5]. In the diabetic heart, because of the lack of insulin function and the usual high level of plasma FAs, glucose use is compromised, and then the heart switches to using FAs exclusively for ATP generation. Consequently, the rate of FAs β-oxidation that occurs mainly in mitochondria is enhanced, FAs uptake exceeds the limited oxidation capacity. In this situation, the accumulation of FAs intermediates leads to lipotoxicity in myocardium [6], [7], [8]. Worse, high FAs oxidation increases the oxygen demand and the generation of reactive oxygen species (ROS), which induces oxidative stress, mitochondrial dysfunction, and cell death, ultimately leads to cardiac damage [3], [4], [9]. Besides increased circulating FAs and impaired insulin pathways, the augmented PPARα/PGC1α signaling pathway, which is activated by FAs and FA derivatives, has been also identified as the primary inducer of high FAs oxidation in the diabetic heart [10], [11], [12]. In the diabetic condition, overexpression of PPARα results in enhanced FAs oxidation through activating the expression of related genes.

Acyl-CoA thioesterase 1 (ACOT1) is a key cytosolic enzyme participate in FAs metabolism, hydrolyzing long-chain acyl-CoAs in the cytosol to free FAs and CoA-SH. ACOT1 is also suggested to be a target gene of PPARα [13], [14], [15]. Previous studies showed that the expression profiles of ACOT isoforms changed dramatically during differentiation of cultured rat brown adipocytes [16]. Moreover, upregulation of ACOTs can be induced by high-fat diet in the heart and skeletal muscle of rats [17]. However, the effects and underlying mechanisms of ACOT1 in diabetic heart remain unknown. Here, we studied the role of ACOT1 in myocardia of db/db mice, an animal model of type 2 diabetes, compared with normal control C57BL/Ks. We also investigated whether and how ACOT1 affected cardiac function in diabetes via negative feedback of activated FAs oxidation.

## Materials and Methods

### Ethics Statement

This study was carried out in strict accordance with the recommendations in the Guide for the Care and Use of Laboratory Animals of the National Institutes of Health. The protocol was approved by the Committee on the Ethics of Animal Experiments of the Animal Research Committee of Tongji College (Permit Number: S243).

### Reagents

Chemicals were obtained from (Sigma-Aldrich Shanghai Trading Co Ltd, Shanghai, China) unless otherwise noted. Cell culture medium and fetal bovine serum (FBS) were obtained from Invitrogen (Life Technologies Corporation, Carlsbad, CA). siRNA-Acot1 and the control siRNA-random were synthesized by Ribobio Corporation (Guangzhou, China), based on the sequence of mouse acot1. Mouse monoclonal antibody against β-actin, goat polyclonal antibody of ACOT1, and rabbit polyclonal antibodies against PPARα, PGC-1α, PKCε, PKCβII, and GLUT4 were purchased from Santa Cruz Biotechnology, Inc. (Santa Cruz, CA). Rabbit polyclonal antibody against UCP3 was purchased from Abcam Inc. (Cambridge, MA). Horseradish peroxidase-conjugated secondary antibodies (goat anti-mouse IgG) were purchased from Pierce, Inc. (Thermo Fisher Scientific Inc., Rockford, IL). DNA ladders, prestained protein markers, and protein extraction kit were purchased from Fermentas Inc. (Thermo Fisher Scientific Inc., Glen Buenie, MD). Polyvinylidene difluoride (PVDF) membranes were obtained from Millipore (Merck KGaA, Darmstadt, Germany). All other reagents were purchased from standard commercial suppliers, unless otherwise indicated.

### Animals

All surgery was performed under sodium pentobarbital anesthesia, and all efforts were made to minimize suffering. Anaesthetization of rats and mice was preformed with intraperitoneal injections of a xylazine (5 mg/kg) and ketamine (80 mg/kg) mixture, placed in a supine position before they were sacrificed. To assess the adequacy of anesthesia during echocardiographic and hemodynamic examinations, parameters such as responsiveness, blood pressure, respiratory rate and heart rate were monitored. Then they were sacrificed by CO_2_ inhalation. For microarray analysis, six male db/db mice (C57BL/Ks background) and six normal control mice C57BL/Ks (Model Animal Research Center of Nanjing University, China) were studied between the ages of 12 and 14 weeks. For in vivo experiments of ACOT1, we divided the 8-week Sprague Dawley (SD) rats (Animal Care and Use Center of Tongji Medical Collage, HUST) into three groups (control, pcDNA3.1, and pcDNA3.1-Acot1); while the db/db mice (Model Animal Research Center of Nanjing University, Nanjing, China) were randomly divided into five groups (control, pcDNA3.1, pcDNA3.1-Acot1, pSilencer-random and pSilencer-Acot1) at the age of 12 weeks. Both the rats and mice were maintained with 12-h light/12-h dark photoperiods with free access to water and food, and were injected with corresponding plasmids (5 mg/kg) via tail vessels, every other week. Rats (n = 7 per group) and mice (n = 6 per group) were sacrificed at 16 weeks and tissue samples were snap-frozen in liquid nitrogen or collected for paraffin embedding.

### Microarray Analysis for Gene Expression

Heart tissue samples of mice (n = 6 per group) were sent to CapitalBio (Beijing, China) for analysis by oligonucleotide microarrays. The resultant image files were scanned using the LuxScan 3.0 program, and the data for different groups were analyzed using the cluster 3.0 program.

### Quantitative Real-time PCR Analysis

Total RNA from animal hearts were isolated using TRIzol (Invitrogen, Life Technologies Corporation) and reverse-transcribed by PrimeScript RT-PCR Kit (Takara Bio Co. Ltd. Dalian, China). qRT-PCR was performed using SYBR® Premix Ex Taq™ (Takara Bio Co. Ltd. Dalian, China), according to the manufacturer’s protocol with the Rotor-Gene 6000 system (Corbett Life Science, QIAGEN, Hilden, Germany). GAPDH or 18S was used as the housekeeping reference gene, and the relative gene expression was normalized to GAPDH or 18S. Each reaction was performed in triplicate, and analysis was performed by the 2^−ΔΔCt^ method, as described previously [18].

### Plasmid Preparation

Full-length acot1 genes cloned from rat and mouse cardiomyocytes by RT-PCR were ligated into the expression vector, pcDNA3.1 (+) (Invitrogen, Life Technologies Corporation), and the sequences were blasted with rattus and mouse acot1 gene sequences on NCBI with 100% fitness. For expression of siRNA-Acot1 and siRNA-random, two pairs of complementary oligonucleotides were designed based on their sequences, synthesized, annealed, and ligated into p*Silencer* 4.1-CMV neo vector (Ambion, ABI, Austin, TX), according to the manufacturer’s protocol. All nucleotide sequences of the constructed plasmids were confirmed by DNA sequencing analysis. Plasmid DNA was prepared for injection with E.Z.N.A Endo-free Plasmid Maxi Kit (Omega Bio-Tek, Norcross, GA), according to the manufacturers’ protocol.

### Cell Culture and Treatment

H9c2 cells were obtained from the American Type Tissue Collection, and were routinely cultured in Dulbecco’s Modified Eagle Medium with high glucose, L-glutamine, and 10% FBS at 37°C in an atmosphere of 5% CO_2_. The cells were cultured in 6- or 96-well plates and grown to approximately 80% confluence before transfection. The transfection of pcDNA3.1 and pcDNA3.1-Acot1 was performed with Lipofectamine LTX reagent (Invitrogen, Life Technologies Corporation), and the transfection of siRNA-random and siRNA-Acot1 was with Lipofectamine 2000 reagent (Invitrogen, Life Technologies Corporation), according to the manufacturer’s instructions. Plasmid DNA for transient transfection was prepared with the TaKaRa MiniBEST Plasmid Purification Kit (Takara Bio Co. Ltd., Dalian, China). The cells were harvested 48 h after transfected with plasmids or siRNAs. H9c2 cells were treated with high glucose and palmitate as previous described [19], [20]. H9c2 cells were incubated in DMEM media (normal glucose: 5 mM, high glucose: 30 mM) with 0.25% BSA alone (control) or 250 µM palmitate complexed to 0.25% BSA for 24 h.

### Western Analysis

Protein samples from cell and animal tissues lysates (15–20 µl) were separated by SDS-PAGE (Bio-Rad) electrophoresis using a 10% (wt/vol) acrylamide gel, and were transferred to a PVDF membrane (Millipore, Merck KGaA). After blocking nonspecific sites in 5% nonfat milk, protein blots were incubated with primary antibodies at dilutions of 1∶1000 in blocking solution, then were washed followed by incubation with a peroxidase-conjugated secondary antibody at dilutions of 1∶5000 and 1∶10000 in blocking buffer. The bands were visualized with the enhanced chemiluminescence kit (Pierce, Rockford, IL, USA), according to the manufacturer’s recommendations. Western blots were analyzed by densitometry using ImageJ (National Institutes of Health software).

### Cell Viability Assays

Sulforhodamine B (SRB) was used to indirectly assess cell viability by staining total cellular protein, as described previously [21].

### Cell Apoptosis Assays

AnnexinV/PI staining was used to determine cell apoptosis by flow cytometry, as follows: 48 h after each transfection, as described above, the cells in 6-well plates were harvested and resuspended in binding buffer, and incubated with FITC-conjugated Annexin V and propidium iodide (Annexin V-FITC Apoptosis Detection Kit, BD, San Jose, CA), according to the manufacturer’s protocol, and then analyzed on a FACStar-Plus flow cytometer (BD, Franklin Lakes, NJ). To exclude necrotic cells, only cells with Annexin V-positive and propidium iodide-negative staining were counted for early stages of apoptosis.

### Histological Analysis and Cardiomyocyte Size Measure

After sacrificed, mouse hearts were collected for paraffin embedding. To detect expression level of ACOT1, tissue sections were stained with antibody against ACOT1. The sections were then visualized by light microscopy and photographed (200X), and were measured by the computer-assisted morphometry (Image-Pro Plus Version 6.0). To measure cardiomyocyte size, tissue sections were visualized by light microscopy (400X) after hematoxylin-eosin staining, and the Image J program was used to measure the area of each cell, as adapted from published methods [20], [22], [23]. Over 200 cardiomyocytes were measured per section.

### Analysis of Oxidant Stress

H_2_O_2_, concentration of malondialdehyde (MDA), total anti-oxidation capacity (T-AOC), and total Superoxide dismutase (T-SOD) activity were detected using the corresponding kits (Jiancheng Bioengineering Institute, Nanjing, China), according to the instructions. 2,7-Dichlorodihydrofluorescein diacetate (DCFH-DA) was purchased from Sigma-Aldrich (St. Louis, MO). It was used as an ROS-capturing reagent as described previously [24]. After staining, cells were incubated with 10 µM DCFH-DA at 37°C for 20 min. DCF fluorescence was detected by flow cytometry.

### Echocardiography

After anesthetization, echocardiographic examination was performed on db/db mice before sacrifice, using a high-resolution imaging system with a 30-MHz high frequency scanhead (VisualSonics Vevo770, VisualSonics Inc. Toronto, Canada) [25]. Parameters were obtained as described below. Generally, a parasternal long-axis B-mode image was acquired with appropriate positioning of the scan head so that the maximum left ventricular (LV) length could be identified. Then a clockwise 90° rotation at the papillary muscle level depicted the parasternal short-axis view. From this view, an M-mode cursor was positioned perpendicular to the anterior and posterior walls of the LV, and M-mode image loops were obtained for measurement of wall thickness and chamber dimensions. Each of these captured image loops included 11 to 20 cardiac cycles, and data were averages from at least 3 cycles per loop. Fractional shortening was calculated as follows: FS = (LVDd-LVDs)/LVDd*100% (LVDd, left ventricular diastolic dimension; LVDs, left ventricular systolic dimension). Left ventricular ejection fraction (LVEF) was calculated as follows: LVEF = (LVEDV-LVESV)/LVEDV*100% (LVEDV, left ventricular end-diastolic volume; LVESV, left ventricular end-systolic volume).

### In vivo Hemodynamics

After anaesthetization, LV catheterization was performed on db/db mice before sacrifice. Briefly, a catheter manometer (Millar 1.4F, SPR 835, Millar Instruments, Inc. Houston, TX) was inserted in the right carotid artery and advanced into the left ventricle to measure instantaneous intraventricular pressure and volume. After stabilization, steady-state measurements were recorded. PVAN software (Millar Instruments) was used to perform the cardiac pressure-volume analysis. All data were averages of at least five measurements, each measurement concluded at least ten successive loops. Global systolic function was measured as peak systolic pressure (Pmax), end systolic pressure (Pes), peak instantaneous rate of left ventricular pressure increase and decline (dP/dtmax, dP/dtmin).

### Parameters of Serum Metabolites

Blood samples from all rats and mice were collected, centrifuged at 3000 rpm for 7 min, and the resulting serum levels of glucose, cholesterol, triglycerides, LDL, and HDL were measured on an AEROSET Clinical Chemistry System (Abbott Laboratories).

### Activity of ATPase of Mitochondria

Mitochondria were isolated from mice heart using Mitochondria Isolation Kit (Keygen Biotech Co, Nanjing, China), according to the manufacturer’s protocol. KeyGen ATPase Test Kit (Keygen Biotech Co, Nanjing, China) was used to determine the activity of Na^+^/K^+^-ATPase and Ca^++^/Mg^++^-ATPase of cardiac mitochondrial, according to the instructions.

### Statistic Analysis

All data are presented as mean ± SEM. The Wilcoxon test, the Student’s t test, and ANOVA were performed, to determine statistically significant differences among treatment groups, as appropriate. In all cases, a value of p<0.05 was considered to be statistically significant.

## Results

### Expression of ACOT1 was Upregulated in Hearts of db/db Mice

**Figure 1:**
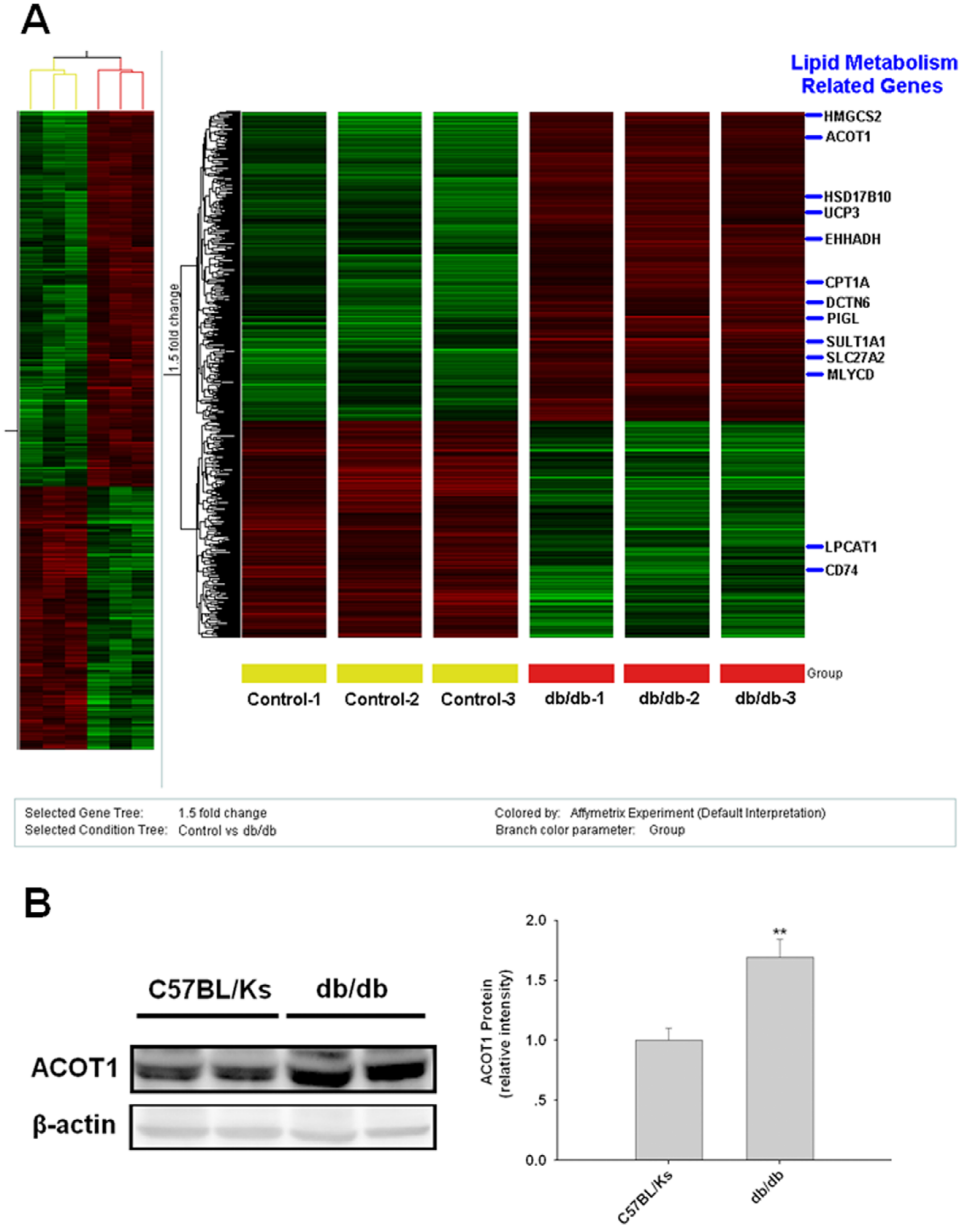
Expression of ACOT1 is upregulated in hearts of db/db mice. (**A**) Partial heat map of upregulated and downregulated gene profiles; 6 db/db mice vs 6 control C57BL/Ks mice were examined. Gene expression levels related to cardiac lipid metabolism compared with the control C57BL/Ks mice are labeled. Eleven genes were detected as selectively upregulated, and 2 genes were detected as selectively downregulated. Red indicates high expression and green indicates low expression. (**B**) Expression of ACOT1 was confirmed by western blot analysis.

To systematically analyze the metabolic changes in the heart tissue of diabetic model, we compared relative gene expression levels in cardiac tissue of db/db mice with normal control C57BL/Ks mice by microarray assays. As shown in Figure 1A, several notable clusters of expression were changed. 13 genes involved in lipid metabolism exhibited differential expression significantly: HMGCS2, HSD17B10, UCP3, EHHADH, CPT1A, DCTN6, PIGL, SULT1A1, SLC27A2, MLYCD and ACOT1 were increased; LPCAT1 and CD74 were repressed. Notably, ACOT1 was dramatically upregulated approximately 11- to 12-fold in db/db mice (Figure 1A), and was confirmed by western blot (Figure 1B). These results were consistent with an earlier study, which showed that ACOT1 expression in the rodent heart was upregulated by FAs and insulin [26]. However, the functions of ACOT1 in heart tissue are still not fully understood.

### ACOT1 Protected Cardiomyocytes from Apoptosis

**Figure 2:**
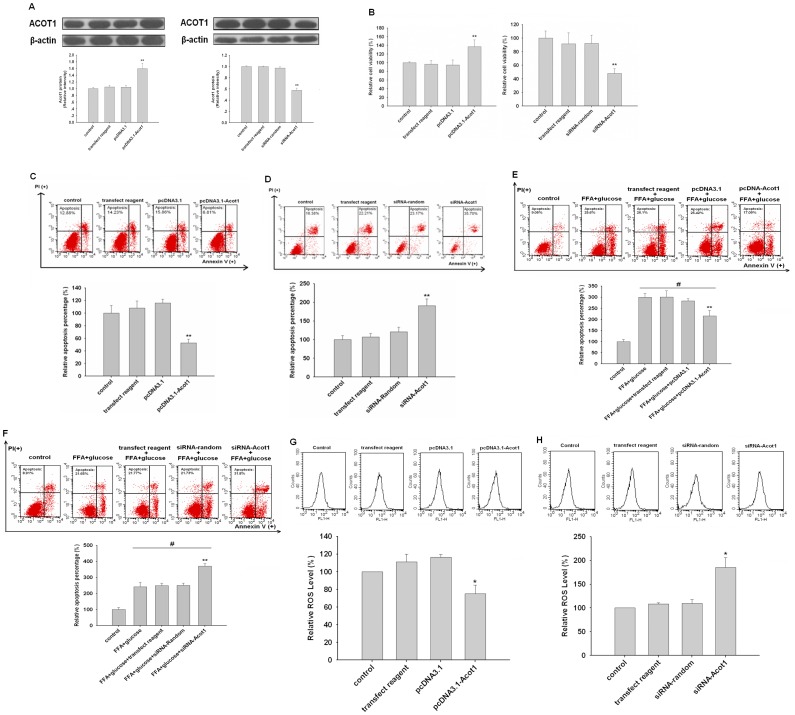
ACOT1 protects cardiomyocytes from apoptosis and enhances its proliferation in vitro. (**A**) H9c2 cells were transfected with pcDNA3.1-Acot1 (1 mg/ml), siRNA-Acot1 (100 nM) for 48 h, and protein expression of ACOT1 was confirmed by western blot. (**B**) Cell survival of treated H9c2 cells as determined by SRB assays. (**C and D**) Cell apoptosis of treated H9c2 cells as determined by Annexin V/PI flowcytometry assays. (**E and F**) Cell apoptosis measured by Annexin V/PI flowcytometry assays after treated with high glucose (30 mM) and palmitate (250 µM) for 24 h in H9c2 cells. (**G and H**) Cell ROS levels of treated H9c2 cells as determined by flow cytometric histogram of DCF fluorescent levels. Similar results were observed in three independent experiments. Data are expressed as relative percentage compared with normal control, ^#,^ *p<0.05 versus control, **p<0.01 versus control.

Considering the enzymatic function of ACOT1 in lipid metabolism, we investigated whether ACOT1 affect the apoptosis of cardiomyocytes. We transfected pcDNA-Acot1 or siRNA-Acot1 into H9c2 cells to upregulate or downregulate ACOT1, respectively, as verified by western blot (Figure 2A). SRB assays showed that overexpression of ACOT1 increased the viability of H9c2 cells, while silencing ACOT1 decreased cell viability (Figure 2B). Next, Annexin V/PI staining assay showed that overexpression of ACOT1 protected H9c2 cells from apoptosis (Figure 2C), while ACOT1 knockdown increased apoptosis (Figure 2D). We further used high glucose and the saturate FA palmitate to induce apoptosis in H9c2 cells, imitating the heart condition of diabetes. Consistently, overexpression of ACOT1 significantly decreased apoptosis, and silencing of ACOT1 resulted in increased apoptosis (Figure 2E and 2F). Since ROS plays a critical role in cell apoptosis, we employed DCFH-DA to examine the ROS production. Significantly, ROS was reduced by over-expression of ACOT1 (Figure 2G). Conversely, silenced ACOT1 lead to high ROS production (Figure 2H). Hence, these data suggested that ACOT1 protected cardiomyocytes from apoptosis, indicating that ACOT1 was a beneficial factor in cardiomyocytes.

### ACOT1 Decreased ROS in Rodent Hearts

**Figure 3:**
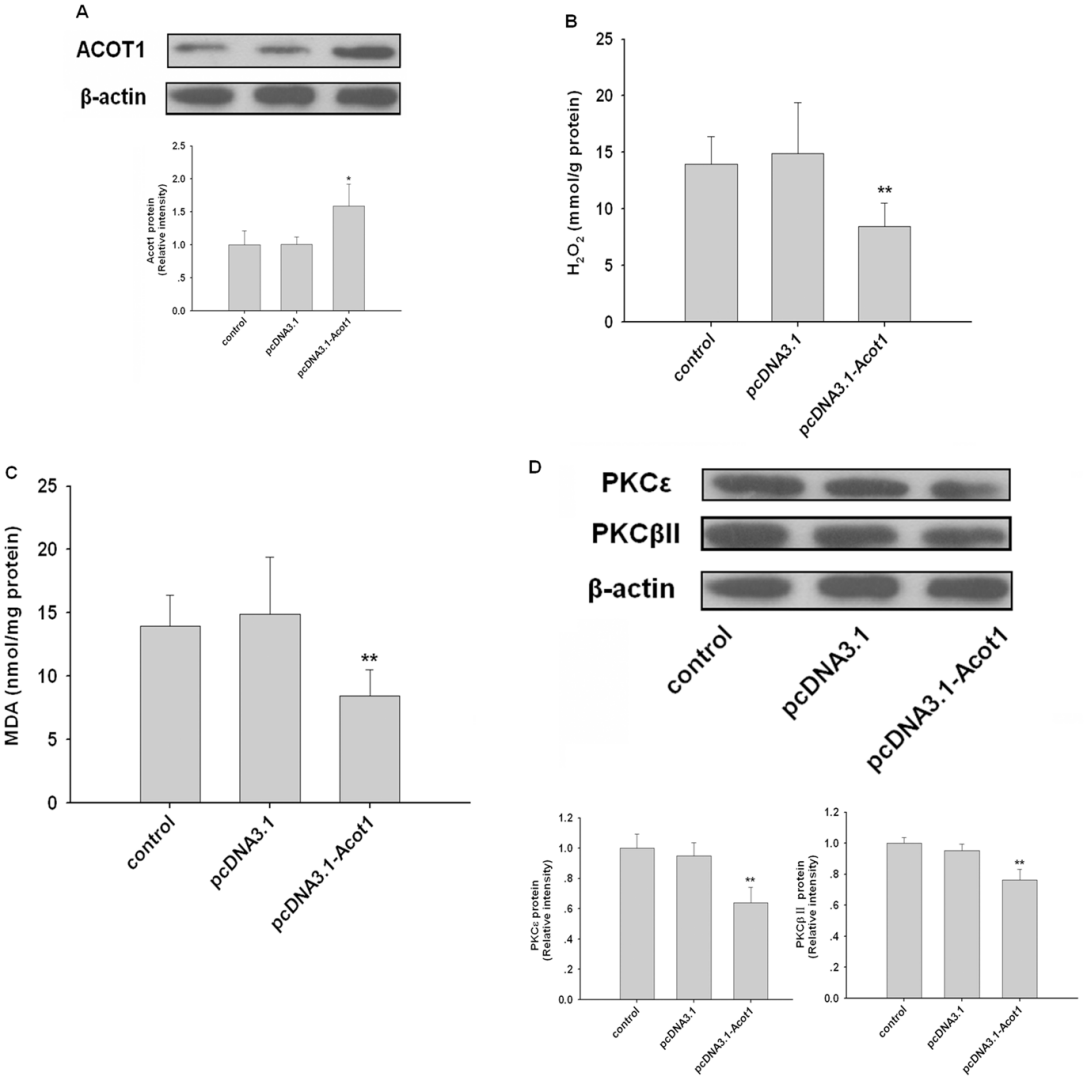
ACOT1 decreases ROS in rodent heart. (**A**) SD rats were injected with corresponding plasmids (control, pcDNA3.1, and pcDNA3.1-Acot1; 5 mg/kg) via tail vessels every other week. After SD rats were sacrificed at 16 weeks, cardiac expression of ACOT1 was confirmed by western blot. (**B and C**) H_2_O_2_ and MDA levels in cardiac tissue of SD rats. (**D**) Cardiac protein levels of PKCε and PKCβII in SD rats as determined by western blot. n = 7, *p<0.05 versus control, **p<0.01 versus control, data are representative of three experiments.

Over the last few decades it has become clear that low levels of ROS play critical roles in cellular homeostasis and cell signaling in the heart [27], [28]. We wondered whether ACOT1 participate in regulation of cardiac ROS generation. Therefore, we injected SD rats with pcDNA3.1-Acot1 to establish an ACOT1-overexpression animal model (Figure 3A). As expected, overexpression of ACOT1 decreased the cardiac H_2_O_2_ generation (Figure 3B) and the levels of MDA in heart (Figure 3C), indicating that ACOT1 could reduce cardiac ROS mostly arising from mitochondria. Furthermore, we found that overexpression of ACOT1 decreased expression of PKCε and PKCβII (Figure 3D), which played key roles in ROS generation. In addition, we also measured the serum free fatty acids (FFAs); the results showed that increased ACOT1 expression didn’t result in significant changes in plasma FFA levels (data not shown). These data suggested that ACOT1 reduced the ROS generation in myocardium.

**Figure 4:**
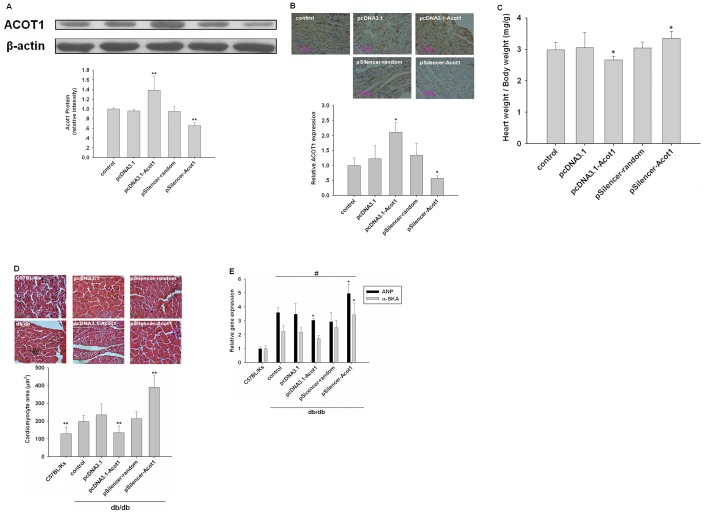
ACOT1 attenuates cardiac dysfunction in db/db mice. (**A and B**) Db/db mice were injected with corresponding plasmids (control, pcDNA3.1, pcDNA3.1-Acot1, pSilencer-random, and pSilencer-Acot1; 5 mg/kg) via tail vessels every other week. After db/db mice were sacrificed at 16 weeks-old, cardiac expression of ACOT1 was confirmed by western blot and immunohistochemical detection (200X magnification). (**C**) Ratio of heart/body weight of db/db mice. (**D**) Cardiomyocyte size visualization and quantitation after different treatment. (**E**) ANP and α-SKA mRNA expression in myocardium of different treated mice measured by real-time PCR. Data are expressed as mean ± S.E., n = 6, *p<0.05 versus control, **p<0.01 versus control, ^#^p<0.05 versus C57BL/Ks, data are representative of three experiments.

### ACOT1 Attenuated Cardiac Dysfunction in db/db Mice

Although was upregulated in diabetic heart, the underlying mechanisms of ACOT1 involved in the regulation of myocardial lipid metabolisms remained to be elucidated. Db/db mice developed severe type 2 diabetes by 8 weeks of age, and cardiac efficiency was remarkably reduced at 15 weeks of age. By modifying the expression of ACOT1, we further explored the effects of ACOT1 on diabetic heart in db/db mice (Figure 4A and 4B). Using Millar cardiac catheter system, we found that ACOT1 overexpression alleviated cardiac dysfunction of diabetes, while deterioration of cardiac deficits was observed when ACOT1 was downregulated by siRNA delivery system (Table 1). Consistent with this in vivo cardiac function analysis using the pressure-volume relationship, the echocardiographic assessment showed that overexpressed ACOT1 induced moderation of left ventricular hypertrophy (LVH) compared with untreated db/db mice. In contrast, silenced ACOT1 aggravated the severity of LVH (Table 2). In addition, increased body weight and heart weight in db/db mice were not changed by ACOT1 treatment (Figure S1A and S1B), but ACOT1 significantly decreased the ratio of heart weight to body weight, and ACOT1 deficiency resulted in an elevated ratio (Figure 4C), indicating the protective role of ACOT1 in diabetic LVH. Also, the anti-hypertrophy effects of ACOT1 were confirmed by myocyte size measurement (Figure 4D) and real-time PCR assays of ANP and α-SKA, consistently (Figure 4E). Then we found that blood glucose, cholesterol and FFAs were not affected obviously by administration of ACOT1 in db/db mice (Table S1), which suggested that ACOT1 protects diabetic hearts from dysfunction not through changing blood FFAs, but some other molecular mechanisms.

These results suggested that ACOT1 overexpression attenuates the cardiac dysfunction of diabetes, while its deficiency leads to aggravation of LVH.

**Table 1:** Comparison of hemodynamic variables for control, pcDNA3.1, pcDNA3.1-Acot1, pSilencer-random, and pSilencer-Acot1 mice.

	C57BL/Ks	db/db	db/db+pcDNA3.1	db/db+pcDNA3.1-Acot1	db/db+pSilencer-random	db/db+pSilencer-Acot1
HR (bmp)	328±12*	304±15	307±12	317±11	310±10	290±19
dP/dt_max_ (mmHg/s)	8321±335**	5312±503	5338±287	6968±672**	5117±253	4387±463*
dP/dt_min_ (mmHg/s)	−7645±392**	−5354±366	−5102±538	−6386±448**	−5170±607	−4284±667*
P_max_ (mmHg)	123.3±13.6**	83.7±5.3	85.2±9.1	105.4±17.5*	86.1±6.8	66.8±8.0*
P_es_ (mmHg)	116.5±12.2**	81.5±8.2	80.5±8.7	104.8±18.0*	81.2±6.7	61.8±8.6*

Values represent mean±SEM; (*n* ≥5 for each group). *p<0.05 versus db/db, **p<0.01 versus db/db. HR, heart rate; dP/dt_max_, peak instantaneous rate of left ventricular pressure increase; dP/dt_min_, peak instantaneous rate of left ventricular pressure increase decline; P_max_, peak systolic pressure; P_es_, end systolic pressure.

**Table 2:** Echocardiographic characteristics of control, pcDNA3.1, pcDNA3.1-Acot1, pSilencer-random, and pSilencer-Acot1 mice.

	C57BL/Ks	db/db	db/db+pcDNA3.1	db/db+pcDNA3.1-Acot1	db/db+pSilencer-random	db/db+pSilencer-Acot1
HR (bpm)	516±28**	460±21	468±18	507±13**	468±21	431±23*
LVIDs (mm)	1.46±0.05**	1.87±0. 08	1.73±0.16	1.71±0.14*	1.76±0.13	2.15±0.27*
LVIDd (mm)	3.37±0.28	3.46±0.22	3.40±0.14	3.63±0.31	3.49±0.39	3.54±0.61
FS (%)	56.6±2.72**	45.9±4.13	48.9±5.5	52.5±4.9*	48.9±7.0	37.6±4.8*
LVEF (%)	92.1±2.0**	78.1±2.4	76.9±1.5	85.5±3.6**	75.4±4.0	71.5±3.1**

Values represent mean±SEM; (*n* ≥5 for each group). *p<0.05 versus db/db, **p<0.01 versus db/db. HR, heart rate; LVIDs, LV internal diameter at systole; LVIDd, LV internal diameter at diastole; FS, fractional shortening; LVEF, LV ejection fraction.

### ACOT1 Reduced Oxidative Stress in db/db Hearts

**Figure 5:**
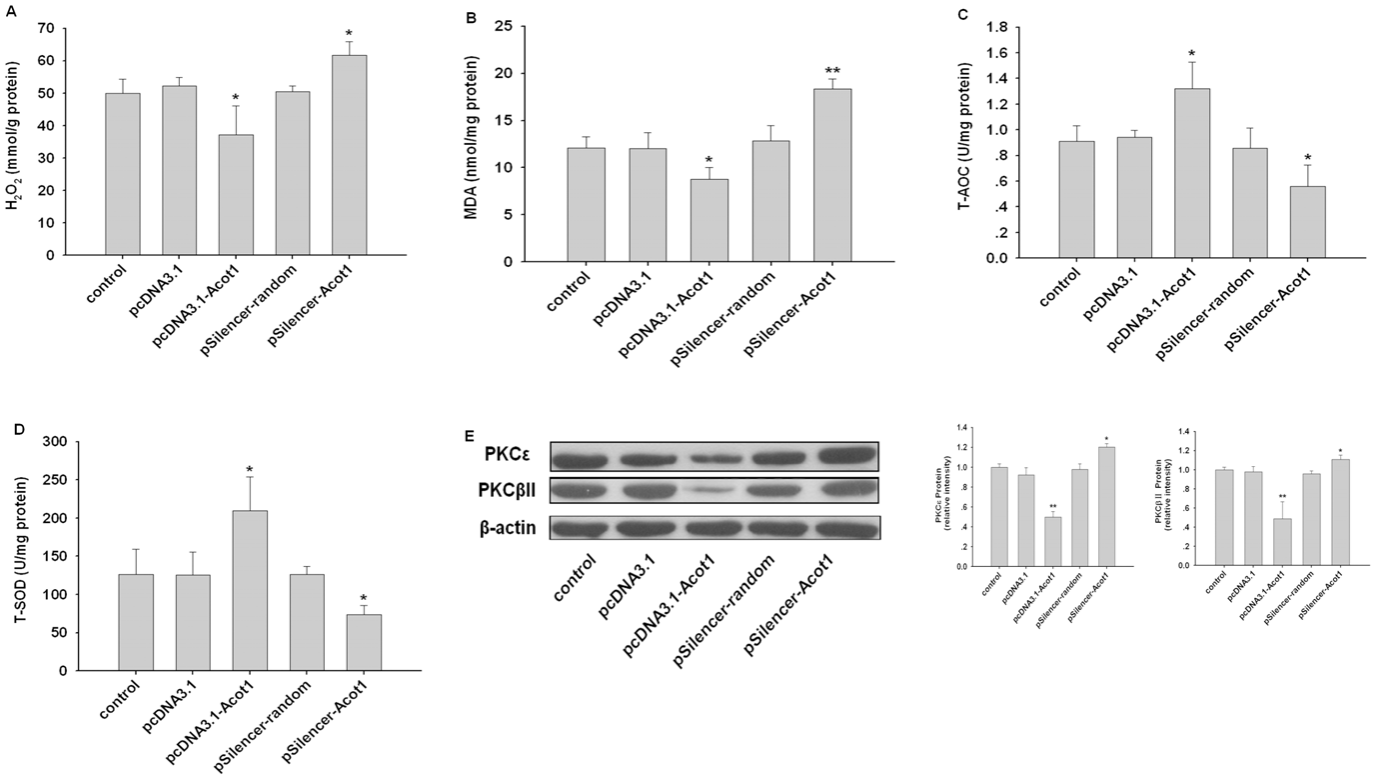
ACOT1 reduces oxidative stress in db/db heart. (**A–D**) Levels of H_2_O_2_, MDA, T-AOC, and T-SOD in myocardium of db/db mice. (**E**) Protein levels of PKCε and PKCβII in myocardium of db/db mice. n = 6, *p<0.05 versus control, **p<0.01 versus control, data are representative of three experiments.

Since we observed that ACOT1 reduced ROS generation in myocytes of SD rats, it was assumed that ACOT1 may reduce ROS damages in diabetic heart through counteracting the increased FAs use. As expected, ACOT1 overexpression remarkably decreased cardiac levels of H_2_O_2_, while silencing ACOT1 increased H_2_O_2_ production (Figure 5A and 5B). On the other hand, increased ACOT1 expression enhanced T-AOC and T-SOD activities, representing its antioxidant capacity (Figure 5C and 5D), which is cardioprotective [28]. In addition, protein levels of PKCs were decreased by ACOT1 overexpression and increased with ACOT1 deficiency (Figure 5E). These results suggested that ACOT1 can reverse oxidative stress damages in diabetic heart, thereby contributing to the cardiac protection in diabetes.

### ACOT1 Reversed Altered Myocardial Energy Substrate Use in Diabetes via Regulation of PPARα/PGC1α Signaling

**Figure 6:**
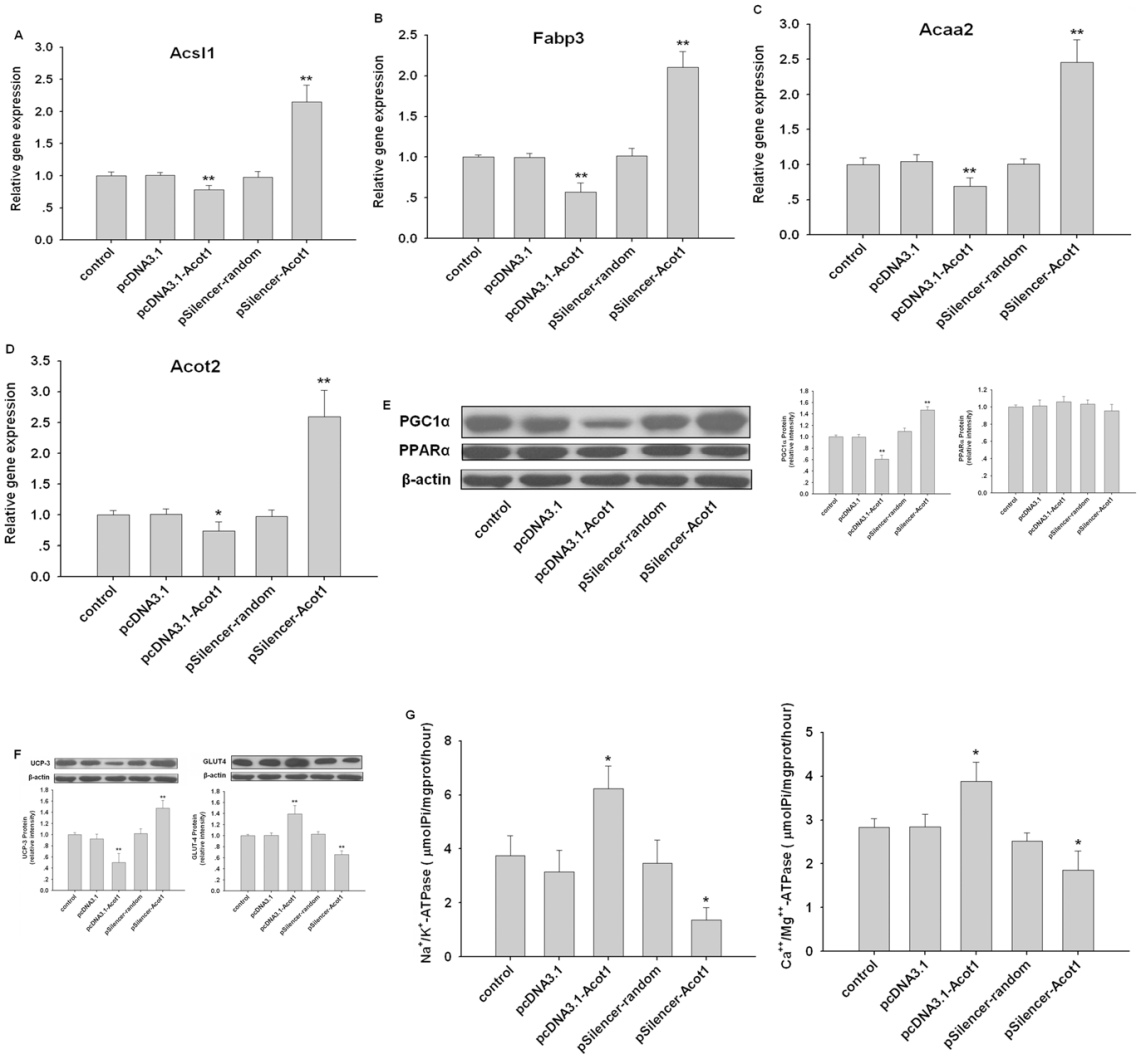
ACOT1 reverse altered myocardial energy substrate use in diabetes. (**A–D**) The expression levels of Acsl1, Fabp3, Acaa2, and Acot2 in myocardium of db/db mice determined by real-time PCR. (**E**) Western blot of PPARα and PGC1α in myocardium of db/db mice. (**F**) The cardiac protein levels of UCP3 and GLUT-4 in db/db mice. (**G**) The activities of Na^+^/K^+^-ATPase and Ca^++^/Mg^++^-ATPase in cardiac mitochondria of db/db mice. Data are expressed as mean ± S.E., n = 6, *p<0.05 versus control, **p<0.01 versus control.

Altered myocardial substrate metabolism, shifting from glucose to fatty acid, has been shown to primarily contribute to diabetic cardiomyopathy [3], [29], [30]. Therefore, by detecting expression of lipid and glucose metabolic genes using real-time PCR in the heart of db/db mice, we investigated the potential molecular modulation mechanisms of ACOT1. We found that ACOT1 overexpression significantly decreased the expression of acsl1, fabp3, acaa2 and acot2, which are all PPARα target genes that participate in fatty acid oxidation (FAO), compared with untreated db/db mice. In contrast, silencing ACOT1 stimulated the expression of these genes (Figure 6A–D). Hence, it was possible that, through the attenuation of enhanced FAO, overexpression of ACOT1 reversed diabetes-induced altered myocardial energy substrate use to ameliorate cardiac dysfunction.

It was notable that Acsl1, Fabp3, Acaa2, and Acot2 are all targets of PPARα, a major transcriptional regulator of lipid metabolism in the heart [12], [31], [32]. We hypothesized that PPARα might be regulated by ACOT1. Western blots showed that protein levels of PPARα were not obviously regulated by ACOT1 (Figure 6E). However, the protein levels of PGC1α were markedly reduced by ACOT1 overexpression, whereas silencing ACOT1 promoted its expression (Figure 6E). In addition, UCP3 was downregulated by overexpression of ACOT1 (Figure 6F). However, the expression of another PPARα target gene responsible for glucose use, GLUT-4, was promoted in response to enhanced ACOT1 expression (Figure 6F). Moreover, the activity of ATPase of cardiac mitochondria was increased by high levels of ACOT1, and suppressed by reduced ACOT1 (Figure 6G). These results suggested that the overexpression of ACOT1 in diabetic heart could reverse the alteration of energy substrate use: on one hand, through reducing the overload of long-chain FA-CoAs for secondary β-oxidation; on the other hand, through the attenuation of enhanced PPARα/PGC1α signaling. This could explain the mechanism by which ACOT1 attenuates cardiac dysfunction in diabetic hearts.

## Discussion

In this study, we have shown that diabetes resulted in enhanced cardiac expression of ACOT1. In the hearts of db/db mice that overexpressed ACOT1, H_2_O_2_ and MDA, which reflected ROS generation arising from mitochondria, were reduced; the activity of ATPases in mitochondria associated with mitochondrial function were promoted; the expression of UCP3 contributing to oxygen wastage for noncontractile purposes was decreased; and cardiac dysfunction was attenuated, as determined by both hemodynamic and echocardiographic detections. LVH was an important hallmark of diabetic cardiomyopathy, as determined by increased cardiomyocyte cross-sectional areas, ANP and α-SKA mRNA levels, which were also confirmed by previous studies [20], [33]. Overexpression of ACOT1 attenuated these LVH indicators as well as whole heart weight and ratio of heart/body weight which represents hypertrophy at the overall level [34], suggesting a protective role of ACOT1. Consistently, ACOT1 deficiency showed opposite effects, which accelerated the cardiac damage induced by diabetes. Notably, we found that overexpression of ACOT1 in diabetic heart repressed the PPARα/PGC1α signaling, as shown by the decreased expression of PGC1α and the downstream genes involved in FA use (Acsl1, Fabp3, Acaa2, and Acot2). These findings suggested that overexpression of ACOT1 could reverse altered myocardial energy substrate use in diabetes via PPARα/PGC1α signaling, thereby ameliorating the myocardium from diabetes-induced damages.

Emerging evidences supported the concept that overuse of FAs, including increased FAs uptake and oxidation, might be the central contributor to the cardiac damages in diabetes, through enhanced oxidative stress induced by augmented ROS generation [35], [36], accumulation of lipids of lipotoxicity [35], [37], [38], mitochondrial dysfunction [3], [9], [39], and decreased cardiac efficiency [9], [39], [40]. Thus, any factor that could counteract enhanced FAs uptake or oxidation might rescue cardiac dysfunction in diabetic hearts.

In the present study, we found that ACOT1 was upregulated in the myocardial tissue of db/db mice, an animal model of type 2 diabetes. This is compatible with previous demonstrations of the upregulation of ACOT1 in hearts of rats chronically fed a high-fat diet [17], [26], [41]. Since enhanced FAs β-oxidation, following the oversupply of long-chain acyl-CoAs in diabetic hearts [41], [42] is attributable to initiation of oxidative stress and finally cardiomyopathy, the hydrolyzation of long-chain acyl-CoAs by ACOT1 is predicted to be an effective inhibition of increased FAs oxidation.

Under normal physiological conditions, mitochondria are the major sources of ROS generation within cardiomyocytes [4], and low level of ROS can be scavenged by antioxidant defenses. When ROS generation exceeds the antioxidant rate, or antioxidant defense is impaired, oxidative stress occurs [28], similar to what occurs in diabetic heart [4], [6]. Recently, it has been shown that because of insulin resistance, excess FAs have to enter the mitochondria for oxidation [43]. Thus, incomplete β-oxidation of FAs, together with high rates of FAs oxidation, increases mitochondrial potential, leading to augmented ROS generation, which induces oxidative stress and cell death [3], [9], [44]. Thus, through reducing long-chain acyl-CoAs (the form that enters mitochondria for β-oxidation), overexpression of ACOT1 may reduce the generation of ROS in the hearts of db/db mice, as we observed. We measured the same effects of ACOT1 on ROS generation in physiological heart of SD rats, which also suggested an important role of FA oxidation in the generation of ROS [45]. In addition to inhibited FAs oxidation, it seemed that enhanced antioxidant ability contributed to the depression of ROS generation. It has been reported that both increased ROS generation and impaired antioxidant defenses contributed to oxidative stress in diabetic hearts [4], and the inactivity of SOD in mitochondria correlates with the generation of superoxide and its related species, which were enhanced under conditions of oxidative stress [46]. Hence, we suggested that through decreasing FA oxidation, ACOT1 ameliorated the inactivity of mitochondrial SOD induced by oxidative stress. It is well known that diabetes-induced ROS could amplify PKCs, which operate a third common pathway that contributes to the development of diabetic cardiomyopathy [11], [47]. Thus, reduced ROS might be a mechanism by which overexpression of ACOT1 decreased the levels of PKCs. It is clear that increased ROS in diabetic hearts, resulting from excessive FAs oxidation, can attack FAs to form lipid peroxides such as MDA, which can also cause mitochondrial damage [6]. This may explain why MDA was reduced under conditions of overexpressed ACOT1.

It is believed that in diabetic hearts, chronically enhanced FA oxidation leads to mitochondrial dysfunction [48]. Indeed, studies have shown that ROS and the accumulation of ROS-induced lipid peroxides take part in the development of mitochondrial defects, including reduced ATP production and mitochondrial calcium uptake [49], which lead to impaired myocardial contractility [3], [4]. Since ATP generated in mitochondria is preferentially used by ion transporter, such as Ca^2+^-ATPase and Na^+^-K^+^-ATPase [50], reduced ATP from increased FA oxidation results in the inactivity of the ATPases [3]. Hence, through inhibition of enhanced FA oxidation in diabetic heart, and thereby, ROS generation, overexpression of ACOT1 could ameliorate mitochondrial dysfunction.

Earlier studies have demonstrated that, compared with glucose use, FA oxidation consumes more oxygen for cardiac ATP production [3], [6]. Hence, FA is an inefficient substrate, and elevation of FA oxidation in diabetic heart results in more myocardial oxygen consumption (MVO_2_) [51], which is defined as the condition of reduced cardiac efficiency (cardiac work/O_2_ consumption). Moreover, this cardiac inefficiency could be accelerated by diabetes-induced mitochondrial uncoupling, a mechanism regulated by UCPs. UCPs contribute to oxygen wastage for noncontractile purposes [9]. Therefore, both hemodynamic and echocardiographic detection of improved cardiac function reflect the recovery of cardiac efficiency in diabetes by overexpression of ACOT1. The initiation of the recovery is still the counteraction of enhanced FA oxidation by ACOT1.

Taken together, we see from its enzymatic function that ACOT1 reduced the overload of long-chain acyl-CoAs, the accumulation of which has been proven to promote the development of insulin resistance and cellular apoptosis [3], [52]. Therefore, ACOT1 inhibited enhanced FA oxidation to exert its cardiac protection.

We were surprised to find that the diabetes-induced PPARα target genes, such as acsl1, fabp3, acaa2, and acot2, were downregulated by overexpression of ACOT1. As the principal regulator in cardiac FAs metabolism, PPARα was confirmed to be over-activated in diabetic hearts, which activate genes involved in various steps of FAs uptake and oxidation [53]. Simultaneously, genes involved in glucose use are downregulated by PPARα [10]. A recent study showed that the cardiac phenotype induced by PPARα overexpression mimics that caused by diabetes mellitus, and this proved the activation of PPARα/PGC1α signaling to be one independent mechanism to contribute to substrate switching and diabetic cardiomyopathy [10]. ACOT1 has been shown to be a target gene of PPARα, which is upregulated by activation of PPARα [13]. However, we found that overexpression of ACOT1 in diabetic hearts could repress the PPARα/PGC1α signaling pathway, as shown by decreased expression of downstream genes involved in FAs use and increased expression of target genes responsible for glucose metabolism. FAs and its derivatives act as ligands for PPARα [12], [54]; hence, higher cardiac PPARα activity is always observed in diabetes. However, a recent study described differing affinities for different ligands that bind PPARα. PPARα binds long-chain acyl-CoAs and unsaturated long-chain FAs with high affinity, but not saturated long-chain FAs [55]. Since saturated FA is the major substrate in animals, this suggests that gene alterations resulting in high levels of acyl-CoAs causes PPARα activation, whereas gene alterations resulting in reduced acyl-CoAs levels inactive PPARα, which is consistent with published reports [55]. Therefore, by reducing the overload of long-chain acyl-CoAs, overexpressed ACOT1 might reduce enhanced PPARα/PGC1α signaling in diabetic hearts. This could be another mechanism to explain why overexpression of ACOT1 could reverse metabolic substrate alteration and protect against diabetic cardiomyopathy.

In conclusion, we have demonstrated that overexpression of ACOT1 reduced the excess long-chain acyl-CoAs for β-oxidation and repressed PPARα/PGC1α signaling pathway to reverse the altered substrate metabolism and attenuate increased oxidative stress, mitochondrial dysfunction, and cardiac inefficiency in diabetic hearts. Therefore, ACOT1 plays a crucial role in diabetic hearts through the regulation of substrate metabolism. These results suggested a new therapeutic target for treatment of cardiac dysfunction related to metabolic diseases, such as obesity and diabetes. However, this observation needs to be confirmed in transgenic MHC-Acot1 mice to explore whether other mechanisms exist in the ACOT1-regulation of cardiac tissues of other pathological models.

## Supporting Information

Figure S1 **Body weight and heart weight in db/db mice.** (A) Body weight of treated mice; (B) Heart weight of treated mice. Data are expressed as mean ± S.E., n = 6, **p<0.01 versus control, data are representative of three experiments.
(DOC)

Table S1 **Physiological parameters of control, pcDNA3.1, pcDNA3.1-Acot1, pSilencer-random, and pSilencer-Acot1 mice.** Values represent mean ± SEM (*n* ≥5 for each group). *p<0.05 versus db/db, **p<0.01 versus db/db.
(DOC)
