# Supplementary material for: Protective Effects of Acyl-coA Thioesterase 1 on Diabetic Heart via PPARα/PGC1α Signaling
Source: PLoS One. 2012 Nov 30;7(11):e50376. doi: 10.1371/journal.pone.0050376 (PMC3511550; doi:10.1371/journal.pone.0050376)
Supplement: Figure S1 — Body weight and heart weight in db/db mice. (A) Body weight of treated mice; (B) Heart weight of treated mice. Data are expressed as mean ± S.E., n = 6, **p<0.01 versus control, data are representative of three experiments. (DOC) [file pone.0050376.s001.doc]

**Figure S1**

**
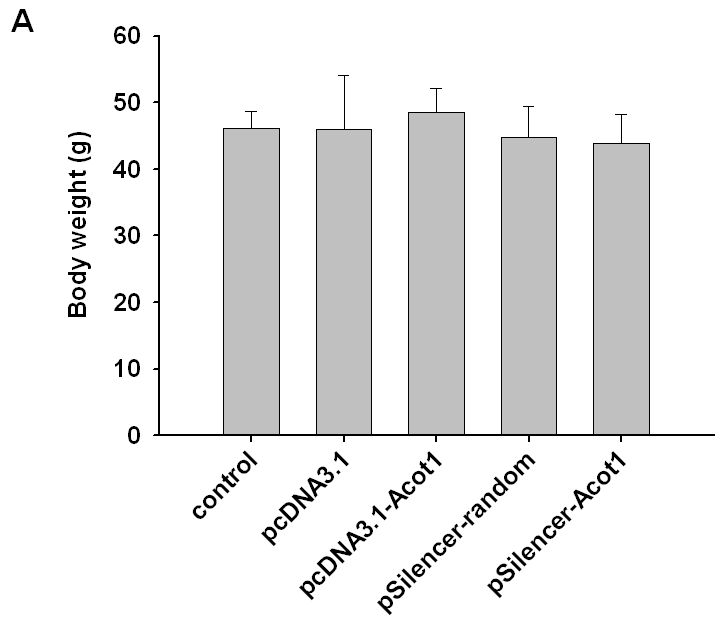
**

**
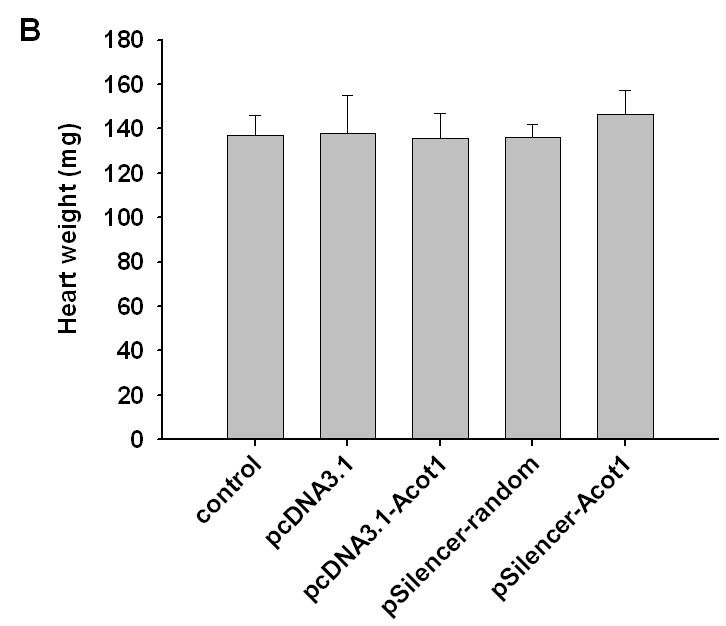
**

**Body weight and heart weight in db/db mice.** (A) Body weight of treated mice; (B) Heart weight of treated mice. Data are expressed as mean ± S.E., n=6, **p<0.01 versus control, data are representative of three experiments.
