# Supplementary material for: Protective Effects of Acyl-coA Thioesterase 1 on Diabetic Heart via PPARα/PGC1α Signaling
Source: PLoS One. 2012 Nov 30;7(11):e50376. doi: 10.1371/journal.pone.0050376 (PMC3511550; doi:10.1371/journal.pone.0050376)
Supplement: Table S1 — Physiological parameters of control, pcDNA3.1, pcDNA3.1-Acot1, pSilencer-random, and pSilencer-Acot1 mice. Values represent mean ± SEM (n ≥5 for each group). *p<0.05 versus db/db, **p<0.01 versus db/db. (DOC) [file pone.0050376.s002.doc]

**Table S1**

**Physiological parameters of control, pcDNA3.1, pcDNA3.1-Acot1, pSilencer-random, and pSilencer-Acot1 mice.**

|  | C57BL/Ks | db/db | db/db+  pcDNA3.1 | db/db+  pcDNA3.1-Acot1 | db/db+  pSilencer-random | db/db+  pSilencer-Acot1 |
| --- | --- | --- | --- | --- | --- | --- |
| **Glucose (mmol/l)** | 10.82±1.62** | 32.45±0.80 | 34.02±7.76 | 33.84±8.60 | 33.14±5.74 | 35.56±7.38 |
| **Cholesterol** **(mmol/l)** | 1.75±0.17** | 2.44±0. 33 | 2.44±0.13 | 2.24±0.28 | 2.36±0.12 | 2.59±0.72 |
| **Free fatty acid (mM)** | 0.43±0.08** | 0.97±0.14 | 1.01±0.10 | 0.91±0.14 | 1.01±0.11 | 1.04±0.07 |

Values represent mean ± SEM (*n* ≥ 5 for each group). *p<0.05 versus db/db, **p<0.01 versus db/db.
